# Mental Health Status of Oncology and Non-Oncology Patients Undergoing Bone Scintigraphy

**DOI:** 10.1192/j.eurpsy.2026.11659

**Published:** 2026-07-31

**Authors:** D. Schneider-Matyka, K. Rachubińska, D. Sałacińska, A. Królikowska, E. Grochans, A. Cybulska

**Affiliations:** 1 Department of Nursing; 2 Scientific Club at the Department of Nursing, Pomeranian Medical University in Szczecin, Szczecin, Poland

## Abstract

**Introduction:**

Bone scintigraphy is a key imaging method in nuclear medicine, widely used to assess oncological and skeletal disorders. Its high sensitivity allows early detection of metabolic bone changes, and advanced techniques such as SPECT/CT enable detailed structural and functional evaluation. Despite its diagnostic value, waiting for scintigraphy results can generate significant stress and anxiety, particularly among oncology patients, thus negatively affecting overall and mental health.

**Objectives:**

This study aimed to compare the mental health status of oncology and non-oncology patients undergoing bone scintigraphy using the GHQ-28 questionnaire and to evaluate the impact of selected sociodemographic variables (gender, age, education, marital status, and employment) on GHQ-28 outcomes.

**Methods:**

The study included 220 oncology and non-oncology patients from Poland undergoing bone scintigraphy. Mental health was assessed with the GHQ-28, which covers four domains: somatic symptoms, anxiety/insomnia, social dysfunction, and depression. Statistical analyses examined the influence of sociodemographic factors and compared outcomes between oncology and non-oncology patient groups.

**Results:**

Significant differences were observed between oncology and non-oncology patients. Non-oncology patients exhibited poorer overall mental health and more pronounced somatic symptoms. Women scored higher on the GHQ-28, indicating greater psychological distress. Among oncology patients, divorced individuals reported higher levels of depression, while those with lower education experienced more severe somatic complaints.

**Conclusions:**

Sociodemographic factors significantly influence mental health in patients awaiting bone scintigraphy results. Special attention should be given to women, divorced individuals, and patients with lower education levels. These findings highlight the importance of individualized psychological and medical interventions and underscore the need for a tailored approach throughout the diagnostic and therapeutic process.

**Disclosure of Interest:**

None Declared

